# The Impact of Respiratory Function Training and Rehabilitation Nursing on the Recovery of Patients With Mycoplasma Pneumoniae Pneumonia

**DOI:** 10.7759/cureus.53461

**Published:** 2024-02-02

**Authors:** Humayun Saleem, Sarosh Khan Jadoon, Amna Akbar, Nisha Hamid Awan, Emama Arshad Abbasi, Javeria Qadeer Abbasi, Soffia Khursheed, Jhanzeb Ahmed, Mohammad Saleem Khan, Sabahat Tasneem

**Affiliations:** 1 Public Health, Health Services Academy, Muzaffarabad, PAK; 2 General Surgery, Combined Military Hospital, Muzaffarabad, PAK; 3 Accident and Emergency, District Headquarter Hospital Jhelum Valley, Muzaffarabad, PAK; 4 Medicine, Combined Military Hospital, Muzaffarabad, PAK; 5 Pathology, Pakistan Institute of Medical Sciences, Islamabad, PAK; 6 General Surgery, Second Affiliated Hospital of Wenzhou Medical University, Wenzhou, CHN; 7 Medicine, District Headquarter Hospital, Kotli, PAK; 8 Public Health, Health Services Academy, Islamabad, PAK

**Keywords:** pneumonia, nursing, mycoplasma pneumoniae pneumonia, respiratory function training and rehabilitation, nano-acupoint sticking

## Abstract

Introduction

The goal of this study was to see how people who had been diagnosed with Mycoplasma pneumoniae pneumonia (MPP) responded to respiratory function training and rehabilitation (RFTR) nursing.

Methodology

A total of 122 patients (five to 12 years of age) diagnosed with Mycoplasma pneumoniae pneumonia (MPP) and refractory Mycoplasma pneumoniae pneumonia (RMPP) using enzyme-linked immunoassay and PCR were included in this study. These patients were hospitalized at a tertiary care hospital from February 2022 to December 2022. Upon admission, they were assigned a numerical identifier based on the order of admission. Subsequently, they were randomly allocated into two equal groups: the observation (OG) and the control (CG), with each group consisting of 61 patients. Nano-acupoint sticking (NAS) therapy along with respiratory function training and rehabilitation (RFTR) nursing interventions were implemented for patients in the OG.

Results

The observed disparities in forced expiratory volume in one second (FEV1), forced vital capacity (FVC), and the ratio of FEV1 to FVC among the patients did not exhibit significant disparity prior to the commencement of treatment (p values of 0.700, 0.105, and 0.829, respectively). There was no significant difference observed in the range of inflammation in the right lung (p=0.523). Inflammation in the left lung and fluid volume in both lungs are statistically different in both groups (p values of 0.001 and 0.000, respectively). The patients in the observation group exhibited a shorter duration of cough and sputum, disappearance of lung sounds, and length of hospital stays (LOS) compared to the other groups, with statistical significance (p<0.05).

Conclusion

Nano-acupoint sticking (NAS) therapy with respiratory function training and rehabilitation (RFTR) in nursing practice has shown enhanced rehabilitation outcomes for individuals diagnosed with Mycoplasma pneumoniae pneumonia (MPP). The present study focuses on the application of NAS therapy in the context of RFTR for individuals diagnosed with MPP.

## Introduction

Mycoplasma pneumoniae (MP) is classified within the taxonomic class Mollicutes. This organism does not have a cell wall structure, it poses a cell membrane instead. Mycoplasma pneumoniae (MP) is composed of prokaryotic cells, exhibiting characteristics that position it within the spectrum between bacteria and viruses [1]. Mycoplasma pneumoniae (MP) is a prominent causative agent of community-acquired pneumonia; Mycoplasma pneumoniae pneumonia (MPP), contributes to a significant proportion of cases, ranging from 10% to 40% in children [2]. These infections have the potential to result in respiratory ailments such as bronchitis and tracheitis, among other consequences beyond the pulmonary system. Although MPP affects children throughout the year, a higher incidence is observed in the autumn and winter seasons [3].

Pneumonia is a prevalent illness in the pediatric population and holds a prominent position as a primary cause of mortality [4]. The condition predominantly manifests during the winter season or period of weather fluctuations, with its primary clinical manifestations encompassing coughing, nasal discharge, elevated body temperature, and discomfort in the lower chest region. If not properly addressed, MPP has the potential to result in severe consequences, including myocarditis, pericarditis, and systemic illness. Azithromycin is a frequently employed contemporary therapeutic intervention in the clinical management of juvenile MPP. This drug has demonstrated efficacy in suppressing the proliferation of viral or bacterial agents within the human body, hence mitigating symptoms such as coughing and difficulty breathing [5]. Nevertheless, the administration of azithromycin has been associated with the occurrence of adverse effects, including abdominal discomfort, loss of appetite, and gastrointestinal disturbances, particularly in pediatric patients. An MPP infection is a prevalent form of pneumonia that can manifest at any time during the year. It constitutes a significant proportion, ranging from 11% to 39% of community-acquired pneumonia cases in children who are admitted to hospitals. Notably, approximately 17% of affected children necessitate hospitalization [6]. Refractory Mycoplasma pneumoniae pneumonia (RMPP) pertains to individuals who have undergone treatment with macrolide antibiotics for one week or longer yet exhibit deterioration in clinical symptoms and a worsening of lung imaging results. The prevalence of RMPP has exhibited a notable escalation since the year 2000, with a recorded infection rate of 14.3% in Asian nations including China, Japan, and Korea in 2008. In 2012, the rate rose to 53.2%, and in 2018, it increased once more to 83.7% [7].

The prevalence of RMPP exhibits a consistent upward trend every year, accompanied by a protracted duration of sickness. Despite the potential alleviation of symptoms with treatment, children afflicted with RMPP still encounter varied levels of respiratory impairment, necessitating remedial intervention [8]. There exist certain challenges in the realm of treatment that necessitate careful consideration. Respiratory function training and rehabilitation (RFTR) is an established technique utilized in clinical practice for pulmonary rehabilitation training. According to previous research [9], implementing appropriate rehabilitation care methods for individuals with MPP can potentially expedite illness recovery and enhance rehabilitation outcomes. Nano-acupoint sticking (NAS) is a therapeutic technique that involves the application of patches to targeted acupoints to prevent and treat various ailments. This method operates through mechanisms such as penetration, reflection, and regulation. In a randomized controlled clinical trial (RCT) conducted by Hong Yao et al. in 2009, NAS therapy was used in children for bronchial asthma. Several acupoints were used based on Chinese medicine principles, including LU1 (Zhongfu), LU9 (Taiyuan), ST36 (Zusanli), and BL13 (Feishu). NAS patches were applied biweekly, with each session lasting for approximately two hours. It offers several benefits, including ease of administration, notable therapeutic efficacy, and few adverse effects [10].

Respiratory function training and rehabilitation (RFTR) is an approach aimed at enhancing the functionality of the respiratory system through the implementation of aerobic exercise. It is beneficial for those diagnosed with MPP. Respiratory function training and rehabilitation (RFTR) interventions have demonstrated the potential to enhance pulmonary ventilation, enhance the endurance and strength of respiratory muscles, and facilitate lung recovery. Enhancing respiratory muscle function and lung ventilation can accelerate the recovery phase for patients with MPP [11]. Nano-acupoint sticking (NAS) has a regulatory role in modulating the body’s internal equilibrium and blood circulation, hence facilitating the process of self-repair within the body. This study looked at how the rehabilitation outcomes of people with MPP changed when NAS and RFTR were used together.

## Materials and methods

The study was conducted at the Abbas Institute of Medical Sciences (AIMS), Muzaffarabad, Pakistan, between February 2022 and December 2022, and the patients were selected through consecutive sampling. Scalex SP 1.0.01 was used to calculate the sample size with a confidence interval of 95%, an expected prevalence of 40%, and an expected loss of 5%. We kept the precision at ±9%, and we obtained a sample size of 121 [12]. We had to make two groups for the study, so 122 patients were enrolled and distributed in two groups of 61 each.

These patients were diagnosed either with Mycoplasma pneumoniae pneumonia (MPP) or refractory Mycoplasma pneumoniae pneumonia (RMPP). These patients were hospitalized, and data was sought after approval from the ethical committee of the hospital and informed permission forms had been duly signed by the families of all participating patients. Upon admission, they were assigned a numerical identifier based on the order of admission. Subsequently, they were randomly allocated into two groups: the observation group (OG) and the control group (CG).

All patients who were enrolled in the study were required to meet the following criteria: (i) individuals who have been diagnosed with Mycoplasma pneumoniae pneumonia (the diagnosis was made by enzyme-linked immunoassay and PCR); (ii) participants who possess normal cognitive abilities and can actively participate in the research; and (iii) individuals who do not have congenital heart and lung problems or congenital airway narrowing. Patients were required to be excluded from the study if they presented any of the following conditions: (i) individuals affiliated with respiratory distress syndrome; (ii) individuals who are undergoing mechanical ventilation; and (iii) individuals with pre-existing severe illnesses like leukemia and congenital cardiac issues. The patients in the control group were administered treatment for pediatric Mycoplasma pneumoniae pneumonia (MPP) utilizing nano-acupoint sticking (NAS). The application of pharmaceutical patches on the lung Feishu and Shénquè acupoints was carried out on patients exhibiting various symptoms, as determined by the diagnostic methods of traditional Chinese medicine (TCM). Participants in the observation group were administered supplementary rehabilitation nursing techniques (RFTR) in conjunction with the NAS therapy. Initially, it was seen that trained rehabilitation nurses at the national level conducted training sessions for the nursing director and nurses with a responsibility level of N2 or above in the respiratory ward, focusing on specialized procedures.

The training encompassed both theoretical and practical examinations to validate operational standards prior to the implementation of clinical RFTR guidance. The theoretical training included the basics of MPP and the principles behind RFTR. Practical training included RFTR techniques, including exercises to improve lung capacity and breathing techniques, along with the use of spirometers. The duration of the training was three weeks. This process was overseen by trained nurses and the nursing head to upload operational quality (no blinding). Additionally, the initial training session for each patient was collaboratively done by the rehabilitation nurse and the nurse in charge. The specialized nurse collaborated with the responsible nurse to create an individualized training program for the patient, which was subsequently modified at regular intervals in response to the patient’s requirements. The responsible nurse, who had undergone the training, subsequently carried out the daily training of the patient. Furthermore, apart from customary activities such as relaxation exercises, diaphragmatic breathing, and pursed-lip breathing [13], the respiratory function training and rehabilitation (RFTR) program incorporated a respiratory training device (known as an incentive spirometer) consisting of three balls. This device offered eight levels of exhalation adjustment and nine levels of inhalation adjustment. The resistance level was incrementally heightened in accordance with individual patient needs, and the training regimen was conducted twice daily, lasting 15 to 20 minutes per session, while adhering to the notion of avoiding patient tiredness or discomfort. Furthermore, to augment the patient’s engagement and adherence to the training regimen, patients were provided with incentives to blow paper scraps, whistles, or flutes during the training session. Throughout the designated treatment period, a group of respiratory exercises were conducted, encompassing activities such as raising the upper arms, expanding the chest, moving the chest, raising the legs, and doing stepping exercises on a daily basis. Each exercise had a duration ranging from two to three minutes, followed by a rest period of two to three minutes where participants closed their eyes and listened to music. The entire session did not exceed a total duration of 20 minutes.

Patients who were discharged but had not achieved full recovery in terms of lung function, ultrasonography, and imaging exams were advised to persist with training until all markers were restored to normal during their outpatient follow-up. During this time frame, the nurses engaged in the act of making telephone calls to promote and motivate individuals to participate in training activities. The measured values and expected ratios of forced expiratory volume in one second (FEV1), forced vital capacity (FVC), and FEV1/FVC% were looked at to see how lung function and pulmonary ultrasonography changed from the time the patient was admitted to the time he or she was sent home. A comparison was made between the alterations in the extent of inflammation and the volume of fluid in both lungs. The study evaluated the duration of cough and sputum, elimination of lung sounds, and length of stay (LOS) among patients belonging to different categories. The data were subjected to analysis using IBM SPSS Statistics for Windows, Version 25 (released 2017; IBM Corp., Armonk, New York, United States). The statistical analysis employed a completely randomized paired-sample t-test to compare continuous variables, while a chi-square test was utilized to assess the association between categorical variables as given in a contingency table. A p-value below the threshold of 0.05 was deemed to be statistically significant.

## Results

In this study, a total of 61 patients were included in the observational group with a mean age of 9.80±1.64 years, consisting of 34 (55.7%) males and 27 (44.3%) females. Among these patients, 32 (52.5%) were diagnosed with MPP (Mycoplasma pneumoniae pneumonia) and 29 (47.5%) were diagnosed with RMPP (refractory Mycoplasma pneumoniae pneumonia). The control group consisted of a total of 61 patients with a mean age of 9.61±1.76 years, with 32 being male and 29 being female. Among these patients, 35 (57.4%) were diagnosed with MPP, and 26 (42.6%) were diagnosed with RMPP (Table 1). There was no significant difference (p>0.05) observed in the general demographic features of patients across both groups (see p-values in Table 2).

**Table 1:** Basic information of patients in both groups MPP: Mycoplasma pneumoniae pneumonia; RMPP: refractory Mycoplasma pneumoniae pneumonia; OG: observation group; CG: control group; SD: standard deviation

Variables		Mean ± SD
Age (OG)		9.80±1.64
Age (CG)		9.61±1.76
		Frequency (Percent)
Gender (OG)	Male	34 (55.74)
	Female	27 (44.26)
Gender (CG)	Male	32 (52.46)
	Female	29 (47.54)
Vaccination (OG)	No	20 (32.79)
	Yes	41 (67.21)
Vaccination (CG)	No	18 (29.51)
	Yes	43 (70.49)
Monthly family income (OG)	< PKR. 100,000/-	56 (91.80)
	≥ PKR. 100,000/-	5 (8.20)
Monthly family income (CG)	< PKR. 100,000/-	50 (81.97)
	≥ PKR. 100,000/-	11 (18.03)
Residence (OG)	Urban	36 (59.02)
	Rural	25 (40.98)
Residence (CG)	Urban	31 (50.82)
	Rural	30 (49.18)
Household type (OG)	Joint Family	40 (65.57)
	Nuclear Family	21 (34.43)
Household type (CG)	Joint Family	43 (70.49)
	Nuclear Family	18 (29.51)
MPP_RMPP (OG)	MPP	32 (52.46)
	RMPP	29 (47.54)
MPP_RMPP (CG)	MPP	35 (57.38)
	RMPP	26 (42.62)

**Table 2:** Comparison of basic characteristics through paired sample t-test OG: observation group; CG: control group; MPP: Mycoplasma pneumoniae pneumonia; RMPP: refractory Mycoplasma pneumoniae pneumonia

	Mean	t-Statistics	Sig. (2-tailed)
Gender (OG) – Gender (CG)	-0.033	-0.389	0.698
Age (OG) – Age (CG)	0.197	0.604	0.548
Vaccination (OG) – Vaccination (CG)	-0.033	-0.424	0.673
Monthly family income (OG) – Monthly family income (CG)	-0.098	-1.625	0.109
Residence (OG) – Residence (CG)	-0.082	-0.843	0.402
Household type (OG) – Household type (CG)	0.049	0.652	0.517
MPP_RMPP (OG) – MPP_RMPP (CG)	0.049	0.490	0.626

There were no significant differences seen in the FEV1, FVC, or ratio of FEV1 to FVC (FEV1/FVC) across the patients’ groups (p > 0.05). Following the intervention, individuals in the OG exhibited an average FEV1 of 81.27±6.4, an average FVC of 93.19±5.89 and an average FEV1/FVC ratio of 87.47±7.94 (see Table 3).

**Table 3:** The measures to predict values of lung function. FEV1: forced expiratory volume in one second (FEV1); FVC: forced vital capacity; and FEV1/FVC%; OG: observation group, CG: control group, SD: standard deviation *Significant p-value (< 0.05)

	OG (mean ± SD)	CG (mean ± SD)	t-value	p-value
FEVI	81.27±6.4	80.76±8.11	0.388	0.700
FVC	93.19±5.89	92.37±6.35	1.646	0.105
FEVI/FVC	87.47±7.94	87.81±10.56	-0.217	0.829
Range inflammation in right lung	30.58±14.14	30.83±13.06	-0.642	0.523
Fluid volume in right lung	4.07± 0.593	4.29±0.51	-3.997	*0.000
Range inflammation in left lung	39.75±18.36	37.47±15.41	3.670	*0.001
Fluid volume in left lung	4.50±0.72	4.37±0.64	10.411	*0.000

**Figure 1:**
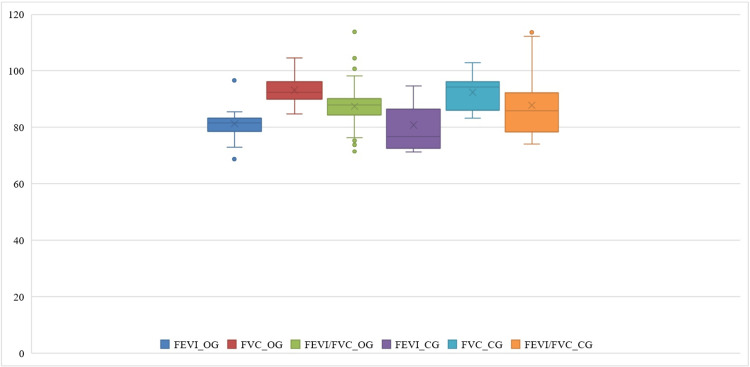
The measures to predict values of related indicators for lung function

**Figure 2:**
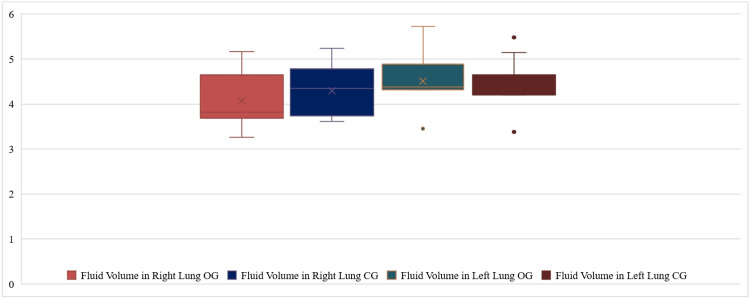
Range of fluid volume (mean volume) of patients in observation group (OG) and control group (CG)

Following various interventions, both cohorts of patients showed enhancement in their FEV1, FVC, and the ratio of FEV1 to FVC; the OG exhibited significantly higher values compared to the CG (Figure 1). A comparative analysis of lung ultrasonography observations among individuals (Table 1) revealed that there were no significant differences (p>0.05) seen in the pre-treatment and post-treatment ranges of inflammation in the right lung in both groups. Fluid volume in the right lung, range of inflammation in the left lung, and fluid volume in the left lung are significantly different in both groups (p<0.05) (Figure 2).

**Figure 3:**
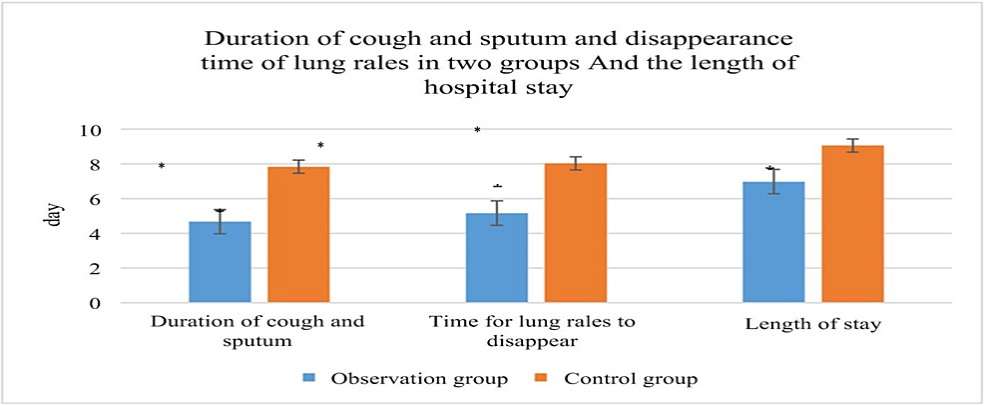
Duration of cough and sputum, disappearance of lung sounds, and LOS of patients after different treatments. Note: * suggested a great difference with P<0.05. It indicates a statistically significant difference. LOS: length of stay

The length of time that a cough and sputum persist, the cessation of lung sounds, and the length of stay of patients: the patients in the OG had a decrease in the duration of cough and sputum, disappearance of lung sounds, and length of stay (LOS), which differed significantly from the patients in the CG (P<0.05), as illustrated in Figure 3.

## Discussion

Acupoints are essentially pores formed where bone, muscle, or fascia intersect, allowing for the passage of neurovascular bundles or nerve-rich connective tissues. They are the sites where the terminal branches of cranial and spinal nerves, along with their accompanying blood vessels, emerge and converge at the central axis of the body in the superficial layer. Additionally, acupoints act as nodes and endpoints connecting the internal zang-fu organs and meridians to the body surface, serving as conduits for the infusion of Qi (vital energy) and blood to the exterior of the body [14]. The Feishu acupoint is a specific acupoint located on the back and has connections with the lungs. It is believed that applying medication to this acupoint can have a direct impact on the lungs, resulting in therapeutic effects for various lung disorders [15].

The Shénquè acupoint has been found to possess the ability to augment the flow of Qi and fortify the physical constitution, thus serving as a preventive measure against relapse. Furthermore, the integration of acupoint stimulation techniques and personalized Chinese medicine formulations has the potential to enhance treatment outcomes by specifically targeting the underlying conditions. The resistance of Mycoplasma pneumoniae pneumonia (MPP) has been observed to increase progressively over the years [13]. The resistant MPP bacteria respond to gamma globulin combined with azithromycin [16]. Concurrently, the prevalence of cases involving MPP combined with other bacterial infections in clinical settings has been on the rise. Consequently, the treatment of MPP has become more challenging and prolonged, posing difficulties in achieving a complete cure. In severe instances, this may result in impairment of lung functions and the development of various pulmonary complications [17,18]. Research has indicated that obstructive ventilation dysfunction is the prevailing pulmonary dysfunction observed in patients with MPP. This dysfunction is closely associated with the adhesion of Mycoplasma pneumoniae (MP) to airway epithelial cells, resulting in damage to the ciliated columnar epithelium, a reduction in the number of cilia, and impaired mucociliary clearance. Additionally, it leads to the obstruction of inflammatory secretion [19].

Furthermore, after infection, the human body may elicit a hyperactive immune response, resulting in the release of a substantial quantity of cytokines (interleukin-1 and IL-6). This excessive cytokine release can lead to an elevation in local mucus production, thereby impacting the normal breathing of children. Patients with refractory Mycoplasma pneumoniae pneumonia (RMPP) exhibit a higher degree of symptoms’ severity, experience a more protracted illness progression, and demonstrate a notable extent of pulmonary impairment [20]. At present, macrolide antibiotics remain the primary treatment option for managing MPP infection. Methods such as nebulization and light percussion can be employed to facilitate pulmonary cleansing and expedite the expulsion of secretions. The integration of rehabilitation nursing principles is necessary in the context of traditional nursing care. Based on the analysis of the pathological alterations following MPP infection and the underlying principles of rehabilitation training technology, it is recommended that a broader range of pulmonary rehabilitation techniques be employed to effectively mitigate clinical symptoms, enhance exercise endurance, and minimize the likelihood of pulmonary complications in pediatric patients [21]. In addition to the procedures that facilitate lung cleansing, it is also possible to employ techniques that enhance lung ventilation, such as pursed-lip breathing and abdominal breathing. Pediatric therapeutic respiratory exercises can incorporate many techniques aimed at enhancing respiratory function, including skeletal muscle activity. The respiratory function training and rehabilitation (RFTR) technique is a sophisticated nursing intervention that effectively promotes the removal of sputum, ensures the openness of the airway, boosts the functioning of respiratory muscles, and optimizes the efficiency of gas exchange. This technique has been extensively studied in the past and proven to be effective [22]. Several studies have shown that RFTR has good clinical results in the context of pulmonary rehabilitation for thoracic surgical conditions, such as chronic obstructive pulmonary disease (COPD), traumatic flail chest, and lung cancer [23,24].

The field of rehabilitation medicine has had significant advancements, leading to the emergence of the concept of “big rehabilitation." This concept has progressively gained more attention, resulting in a transformation of the clinical perspective on illness treatment. The application of RFTR has now been extended to pediatric illnesses. Several studies have utilized RFTR as a therapeutic approach for children with spastic cerebral palsy. These studies have demonstrated that RFTR interventions can lead to improvements in both gross motor function and comprehensive abilities in this population [25,26]. Comprehensive use of RFTR in pediatric patients with spinal deformities has been shown to improve lung function, facilitate smooth surgery, and help patients recover after surgery. Furthermore, there have been investigations conducted on the application of RFTR in the management of acute bronchitis and asthma among pediatric patients. These studies have demonstrated the potential to enhance oxygen pressure levels and facilitate improved lung function [13]. This study implemented a rehabilitation strategy for pediatric patients diagnosed with MPP infection, resulting in favorable clinical outcomes. The results of the study showed that there were differences between the observed and predicted values of forced expiratory volume in one second (FEV1), forced vital capacity (FVC), and the ratio of FEV1 to FVC percentage in pediatric patients when they were discharged. These disparities suggest that RFTR plays a role in the restoration of lung function in children affiliated with MPP.

Limitations

The primary focus of this study was to examine the impact of NAS, in conjunction with RFTR nursing, on the rehabilitation outcomes of individuals diagnosed with MPP. There were certain limitations to the study. It was a single-center study with a small population size. As the treatment is not common in our country, the acceptability was low. Long-term follow-up data for lung function improvement is not available as the last follow-up was done on a telephone call and the patients reported subjective improvement and disagreed to report to the hospital or continue training.

## Conclusions

In conclusion, the integration of NAS and RFTR nursing practices exhibited encouraging rehabilitation outcomes for individuals diagnosed with MPP. The respiratory training approach was characterized by its simplicity, safety, cost-effectiveness, and convenience, as it did not necessitate the use of specialized equipment or impose additional burdens on patients. The intervention demonstrated notable clinical efficacy and holds potential for use in pulmonary rehabilitation across several disciplines and disorders, while also aiding in the amelioration of other symptoms. In this study, a telephone follow-up was undertaken for patients who were released to provide guidance for the continuation of their training. However, it is important to note that statistical data regarding the final follow-up with ultrasound electrodiagnosis was not obtained.

## Appendices

**Table 4:** Data set Basic characteristics (frequencies)

Cases	Male/Female OG	Male/Female CG	Age/OG	Age/CG	Vaccination/OG	Vaccination/CG	MPP / RMPP OG	MPP / RMPP CG	OG/Family Income/month	CG/Family Income/month	Residence OG	Residence CG	Household OG	Household CG
1	2	2	11	9	No	No	RMPP	RMPP	< Rs. 100,000/-	< Rs. 100,000/-	Rural	Urban	Joint Family	Joint Family
2	1	1	8	8	Yes	No	MPP	MPP	< Rs. 100,000/-	< Rs. 100,000/-	Rural	Urban	Joint Family	Joint Family
3	2	2	11	12	Yes	Yes	RMPP	RMPP	< Rs. 100,000/-	< Rs. 100,000/-	Urban	Rural	Nuclear Family	Joint Family
4	1	1	10	8	No	Yes	MPP	RMPP	< Rs. 100,000/-	< Rs. 100,000/-	Rural	Urban	Joint Family	Nuclear Family
5	2	2	11	7	Yes	Yes	RMPP	MPP	< Rs. 100,000/-	≥ Rs. 100,000/-	Rural	Rural	Nuclear Family	Joint Family
6	2	1	8	10	Yes	No	RMPP	MPP	< Rs. 100,000/-	< Rs. 100,000/-	Urban	Rural	Joint Family	Nuclear Family
7	1	2	11	7	Yes	Yes	RMPP	RMPP	≥ Rs. 100,000/-	≥ Rs. 100,000/-	Urban	Urban	Joint Family	Joint Family
8	2	2	10	12	No	No	RMPP	MPP	< Rs. 100,000/-	< Rs. 100,000/-	Rural	Urban	Nuclear Family	Nuclear Family
9	1	1	9	9	Yes	Yes	MPP	RMPP	< Rs. 100,000/-	< Rs. 100,000/-	Urban	Rural	Joint Family	Joint Family
10	1	1	11	8	Yes	Yes	MPP	MPP	< Rs. 100,000/-	< Rs. 100,000/-	Urban	Rural	Nuclear Family	Joint Family
11	1	2	12	10	Yes	Yes	MPP	RMPP	< Rs. 100,000/-	< Rs. 100,000/-	Rural	Urban	Joint Family	Nuclear Family
12	2	1	10	7	Yes	Yes	RMPP	MPP	< Rs. 100,000/-	< Rs. 100,000/-	Urban	Rural	Joint Family	Joint Family
13	1	2	6	9	No	Yes	MPP	MPP	< Rs. 100,000/-	< Rs. 100,000/-	Rural	Urban	Nuclear Family	Joint Family
14	2	2	5	11	No	Yes	RMPP	MPP	< Rs. 100,000/-	< Rs. 100,000/-	Urban	Urban	Joint Family	Nuclear Family
15	1	1	11	7	Yes	Yes	RMPP	RMPP	< Rs. 100,000/-	< Rs. 100,000/-	Rural	Rural	Joint Family	Joint Family
16	2	2	8	12	No	No	MPP	MPP	≥ Rs. 100,000/-	< Rs. 100,000/-	Urban	Urban	Joint Family	Nuclear Family
17	2	1	11	8	Yes	No	RMPP	RMPP	< Rs. 100,000/-	≥ Rs. 100,000/-	Urban	Rural	Joint Family	Joint Family
18	1	2	10	11	Yes	Yes	MPP	MPP	< Rs. 100,000/-	< Rs. 100,000/-	Rural	Urban	Nuclear Family	Joint Family
19	1	2	11	12	Yes	Yes	MPP	RMPP	< Rs. 100,000/-	< Rs. 100,000/-	Urban	Rural	Joint Family	Joint Family
20	2	1	8	11	No	Yes	RMPP	MPP	≥ Rs. 100,000/-	< Rs. 100,000/-	Urban	Urban	Nuclear Family	Joint Family
21	1	2	11	9	Yes	Yes	MPP	MPP	< Rs. 100,000/-	< Rs. 100,000/-	Urban	Urban	Joint Family	Joint Family
22	2	2	10	11	Yes	No	RMPP	MPP	< Rs. 100,000/-	≥ Rs. 100,000/-	Rural	Rural	Nuclear Family	Nuclear Family
23	2	1	9	8	Yes	Yes	RMPP	RMPP	≥ Rs. 100,000/-	< Rs. 100,000/-	Rural	Rural	Joint Family	Joint Family
24	1	1	11	11	Yes	Yes	MPP	MPP	< Rs. 100,000/-	< Rs. 100,000/-	Urban	Urban	Nuclear Family	Nuclear Family
25	2	1	12	10	Yes	Yes	RMPP	RMPP	< Rs. 100,000/-	< Rs. 100,000/-	Urban	Urban	Joint Family	Joint Family
26	1	2	10	11	Yes	Yes	MPP	MPP	< Rs. 100,000/-	< Rs. 100,000/-	Urban	Rural	Joint Family	Joint Family
27	1	2	9	8	No	Yes	MPP	RMPP	< Rs. 100,000/-	< Rs. 100,000/-	Rural	Urban	Joint Family	Nuclear Family
28	1	1	11	11	No	Yes	RMPP	MPP	< Rs. 100,000/-	≥ Rs. 100,000/-	Urban	Urban	Nuclear Family	Joint Family
29	1	2	8	10	Yes	Yes	MPP	RMPP	< Rs. 100,000/-	< Rs. 100,000/-	Urban	Urban	Joint Family	Joint Family
30	2	2	11	11	No	No	RMPP	MPP	< Rs. 100,000/-	< Rs. 100,000/-	Urban	Rural	Joint Family	Joint Family
31	1	1	12	10	No	No	RMPP	RMPP	≥ Rs. 100,000/-	< Rs. 100,000/-	Rural	Rural	Joint Family	Joint Family
32	2	1	11	11	Yes	Yes	MPP	MPP	< Rs. 100,000/-	< Rs. 100,000/-	Urban	Urban	Joint Family	Joint Family
33	1	2	9	8	Yes	Yes	MPP	RMPP	< Rs. 100,000/-	≥ Rs. 100,000/-	Rural	Rural	Nuclear Family	Nuclear Family
34	1	1	11	11	Yes	Yes	MPP	MPP	< Rs. 100,000/-	< Rs. 100,000/-	Urban	Urban	Joint Family	Joint Family
35	1	2	8	10	No	Yes	MPP	RMPP	< Rs. 100,000/-	< Rs. 100,000/-	Urban	Rural	Nuclear Family	Nuclear Family
36	2	1	11	9	No	Yes	RMPP	MPP	< Rs. 100,000/-	< Rs. 100,000/-	Urban	Urban	Joint Family	Joint Family
37	1	1	10	11	Yes	Yes	MPP	RMPP	< Rs. 100,000/-	≥ Rs. 100,000/-	Rural	Urban	Nuclear Family	Nuclear Family
38	2	2	11	12	No	Yes	RMPP	MPP	< Rs. 100,000/-	< Rs. 100,000/-	Rural	Rural	Joint Family	Joint Family
39	1	1	8	10	Yes	Yes	MPP	MPP	< Rs. 100,000/-	< Rs. 100,000/-	Urban	Urban	Joint Family	Joint Family
40	1	1	11	6	Yes	Yes	RMPP	MPP	< Rs. 100,000/-	< Rs. 100,000/-	Urban	Rural	Nuclear Family	Joint Family
41	2	1	10	5	No	No	MPP	RMPP	< Rs. 100,000/-	< Rs. 100,000/-	Rural	Urban	Nuclear Family	Nuclear Family
42	1	2	9	11	Yes	Yes	MPP	MPP	< Rs. 100,000/-	< Rs. 100,000/-	Urban	Urban	Joint Family	Joint Family
43	1	1	11	8	No	Yes	RMPP	RMPP	< Rs. 100,000/-	< Rs. 100,000/-	Urban	Rural	Joint Family	Joint Family
44	1	1	12	6	Yes	Yes	MPP	MPP	< Rs. 100,000/-	≥ Rs. 100,000/-	Rural	Rural	Joint Family	Nuclear Family
45	2	2	10	11	Yes	No	RMPP	MPP	< Rs. 100,000/-	< Rs. 100,000/-	Urban	Urban	Nuclear Family	Nuclear Family
46	1	1	9	8	Yes	Yes	MPP	RMPP	< Rs. 100,000/-	< Rs. 100,000/-	Urban	Rural	Joint Family	Joint Family
47	2	1	8	11	No	No	RMPP	MPP	< Rs. 100,000/-	< Rs. 100,000/-	Rural	Urban	Joint Family	Joint Family
48	1	2	12	10	Yes	Yes	MPP	RMPP	< Rs. 100,000/-	≥ Rs. 100,000/-	Urban	Rural	Joint Family	Joint Family
49	1	1	8	9	Yes	No	MPP	MPP	< Rs. 100,000/-	< Rs. 100,000/-	Urban	Urban	Nuclear Family	Joint Family
50	2	2	7	11	Yes	No	RMPP	MPP	< Rs. 100,000/-	< Rs. 100,000/-	Rural	Rural	Nuclear Family	Nuclear Family
51	1	1	12	12	No	No	MPP	RMPP	< Rs. 100,000/-	< Rs. 100,000/-	Urban	Rural	Joint Family	Joint Family
52	2	2	11	10	Yes	No	RMPP	MPP	< Rs. 100,000/-	< Rs. 100,000/-	Rural	Urban	Nuclear Family	Joint Family
53	1	2	10	9	Yes	Yes	MPP	RMPP	< Rs. 100,000/-	< Rs. 100,000/-	Urban	Rural	Joint Family	Joint Family
54	2	1	9	7	Yes	Yes	RMPP	MPP	< Rs. 100,000/-	< Rs. 100,000/-	Urban	Rural	Joint Family	Nuclear Family
55	2	1	8	11	Yes	No	MPP	RMPP	< Rs. 100,000/-	< Rs. 100,000/-	Rural	Urban	Joint Family	Joint Family
56	1	1	10	8	No	Yes	RMPP	MPP	< Rs. 100,000/-	≥ Rs. 100,000/-	Rural	Urban	Nuclear Family	Joint Family
57	2	2	7	11	Yes	No	MPP	RMPP	< Rs. 100,000/-	< Rs. 100,000/-	Urban	Rural	Joint Family	Nuclear Family
58	2	2	9	10	No	Yes	RMPP	MPP	< Rs. 100,000/-	< Rs. 100,000/-	Urban	Rural	Joint Family	Joint Family
59	1	1	11	9	Yes	Yes	MPP	MPP	< Rs. 100,000/-	< Rs. 100,000/-	Urban	Rural	Nuclear Family	Joint Family
60	1	1	7	11	Yes	Yes	MPP	RMPP	< Rs. 100,000/-	≥ Rs. 100,000/-	Rural	Urban	Joint Family	Joint Family
61	2	2	12	12	Yes	Yes	RMPP	MPP	< Rs. 100,000/-	< Rs. 100,000/-	Rural	Rural	Joint Family	Joint Family

**Table 5:** Data set Variables for lung function

SN	FEVI_OG	FVC_OG	FEVI/FVC_OG	FEVI_CG	FVC_CG	FEVI/FVC_CG	
1	85.41	96.209	88.78	72.62	89.5	81.14	
2	83.32	92.405	90.17	82.6	96.24	85.83	
3	84.6	92.405	91.55	72.62	96.24	75.46	
4	81.6	84.807	96.22	94.6	83.245	113.64	
5	72.9	98.711	73.85	71.26	94.28	75.58	
6	83.32	92.405	90.17	72.62	96.24	75.46	
7	78.56	84.807	92.63	76.65	83.245	92.08	
8	81.6	104.57	78.03	93.45	102.86	90.85	
9	96.56	92.405	104.50	72.62	96.24	75.46	
10	83.32	96.209	86.60	82.6	89.5	92.29	
11	75.6	84.807	89.14	76.65	83.245	92.08	
12	68.75	92.405	74.40	86.4	96.24	89.78	
13	78.56	89.991	87.30	71.26	86.126	82.74	
14	83.32	84.807	98.25	76.65	83.245	92.08	
15	78.8	92.405	85.28	72.62	96.24	75.46	
16	85.41	104.57	81.68	86.42	102.86	84.02	
17	83.32	96.209	86.60	82.6	89.5	92.29	
18	81.6	92.405	88.31	94.6	96.24	98.30	
19	96.56	84.807	113.86	76.65	83.245	92.08	
20	78.56	89.991	87.30	72.62	86.126	84.32	
21	83.32	92.405	90.17	92.15	96.24	95.75	
22	84.6	104.57	80.90	76.65	102.86	74.52	
23	75.6	84.807	89.14	71.26	83.245	85.60	
24	81.6	92.405	88.31	82.6	96.24	85.83	
25	83.32	98.711	84.41	72.62	94.28	77.03	
26	68.75	96.209	71.46	86.42	89.5	96.56	
27	78.56	92.405	85.02	71.26	96.24	74.04	
28	75.6	84.807	89.14	93.45	83.245	112.26	
29	83.32	89.991	92.59	76.65	86.126	89.00	
30	78.8	92.405	85.28	82.6	96.24	85.83	
31	81.6	104.57	78.03	76.65	102.86	74.52	
32	72.9	84.807	85.96	72.62	83.245	87.24	
33	83.32	92.405	90.17	86.4	96.24	89.78	
34	85.41	98.711	86.53	71.26	94.28	75.58	
35	78.8	96.209	81.91	92.15	89.5	102.96	
36	78.56	92.405	85.02	82.6	96.24	85.83	
37	83.32	104.57	79.68	76.65	102.86	74.52	
38	75.6	84.807	89.14	72.62	83.245	87.24	
39	84.6	92.405	91.55	86.4	96.24	89.78	
40	68.75	89.991	76.40	94.6	86.126	109.84	
41	96.56	98.711	97.82	86.42	94.28	91.66	
42	81.6	92.405	88.31	92.15	96.24	95.75	
43	85.41	84.807	100.71	71.26	83.245	85.60	
44	78.8	104.57	75.36	82.6	102.86	80.30	
45	83.32	92.405	90.17	72.62	96.24	75.46	
46	68.75	96.209	71.46	76.65	89.5	85.64	
47	75.6	84.807	89.14	93.45	83.245	112.26	
48	78.56	92.405	85.02	86.4	96.24	89.78	
49	81.6	89.991	90.68	86.42	86.126	100.34	
50	84.6	96.209	87.93	92.15	89.5	102.96	
51	83.32	92.405	90.17	82.6	96.24	85.83	
52	96.56	98.711	97.82	72.62	94.28	77.03	
53	72.9	84.807	85.96	76.65	83.245	92.08	
54	96.56	92.405	104.50	94.6	96.24	98.30	
55	78.8	92.405	85.28	71.26	96.24	74.04	
56	81.6	104.57	78.03	86.42	102.86	84.02	
57	83.32	92.405	90.17	76.65	96.24	79.64	
58	75.6	96.209	78.58	72.62	89.5	81.14	
59	78.56	92.405	85.02	71.26	96.24	74.04	
60	84.6	89.991	94.01	93.45	86.126	108.50	
61	83.32	98.711	84.41	82.6	94.28	87.61	

**Table 6:** Data Set Variables for Lung Volume

Range inflammation in left lung OG	Range inflammation in left lung CG	Fluid volume in right lung OG	Fluid volume in right lung CG	Fluid volume in left lung OG	Fluid volume in left lung CG
12.26	16.48	3.26	3.74	5.72	5.48
68.2	63.7	5.16	5.24	3.45	3.38
55.6	46.75	4.65	4.78	4.32	4.2
34	31	3.68	4.35	5.72	5.48
25.78	16.48	4.12	4.35	4.36	4.26
12.26	16.48	3.26	3.74	4.38	4.32
38.2	43.86	3.82	3.61	3.45	3.38
62	55.26	4.65	4.78	4.36	4.26
25.78	23.95	3.26	3.74	4.32	4.2
34	31	3.68	4.35	3.45	3.38
42.6	36.4	5.16	5.24	4.36	4.26
38.2	43.86	3.26	3.74	4.52	4.48
53	49.16	4.65	4.78	4.38	4.32
34	31	3.68	4.35	4.89	4.65
25.78	23.95	3.82	3.61	5.56	5.14
12.26	16.48	4.52	3.72	4.52	4.48
55.6	46.75	3.68	4.35	3.45	3.38
12.26	16.48	4.65	4.78	4.36	4.26
38.2	43.86	3.68	4.35	3.45	3.38
12.26	16.48	4.12	4.35	4.38	4.32
42.6	36.4	3.26	3.74	5.72	5.48
34	31	3.68	4.35	5.72	5.48
12.26	16.48	4.65	4.78	4.38	4.32
25.78	23.95	3.82	3.61	3.45	3.38
62	55.26	5.16	5.24	4.89	4.65
12.26	16.48	3.68	4.35	4.36	4.26
38.2	43.86	3.26	3.74	5.72	5.48
55.6	46.75	4.65	4.78	3.45	3.38
34	31	4.12	4.35	4.52	4.48
42.6	36.4	3.82	3.61	4.36	4.26
12.26	16.48	4.52	3.72	4.38	4.32
68.2	63.7	3.68	4.35	4.32	4.2
25.78	23.95	3.82	3.61	4.52	4.48
38.2	43.86	3.26	3.74	4.52	4.48
53	49.16	4.65	4.78	3.45	3.38
62	55.26	5.16	5.24	4.36	4.26
12.26	16.48	5.16	5.24	5.56	5.14
34	31	3.68	4.35	4.32	4.2
68.2	63.7	4.12	4.35	4.36	4.26
25.78	23.95	4.52	3.72	4.52	4.48
68.2	63.7	4.12	4.35	5.72	5.48
55.6	46.75	3.68	4.35	3.45	3.38
42.6	36.4	4.65	4.78	4.38	4.32
12.26	16.48	3.68	4.35	4.36	4.26
25.78	23.95	3.82	3.61	4.89	4.65
38.2	43.86	3.68	4.35	5.56	5.14
53	49.16	5.16	5.24	4.89	4.65
34	31	3.26	3.74	4.32	4.2
62	55.26	4.65	4.78	3.45	3.38
68.2	63.7	4.12	4.35	5.72	5.48
12.26	16.48	3.68	4.35	4.38	4.32
25.78	23.95	4.12	4.35	4.89	4.65
62	55.26	4.65	4.78	4.36	4.26
42.6	36.4	3.82	3.61	4.32	4.2
68.2	63.7	3.26	3.74	5.56	5.14
55.6	46.75	3.68	4.35	3.45	3.38
34	31	3.68	4.35	4.89	4.65
62	55.26	4.65	4.78	4.36	4.26
55.6	46.75	4.52	3.72	3.45	3.38
42.6	36.4	3.68	4.35	4.38	4.32
53	49.16	4.52	3.72	5.72	5.48
